# Medicine in Its Relations with Botany—A Chair of Botany in Every Medical School—No. 3

**Published:** 1874-09

**Authors:** W. T. Grant

**Affiliations:** Georgia


### MEDICINE IN ITS RELATIONS WITH BOTANY—A CHAIR OF BOTANY IN EVERY MEDICAL SCHOOL—No. 3.

BY W. T. GRANT, M.D., OF GEORGIA.

I have no doubt that there are many ready to say that it is impossible to construct such a dichotomous analysis of plants as I proposed in my last essay on this subject. I could only concede this point on the ground that sufficient data have not yet been collected to allow of its success; but on no other. I may be too enthusiastic, but I have not a doubt of its ultimate feasibility.

But when will we have the necessary data?—and how will we obtain them? It is quite certain that we will never get them by sitting idly down and awaiting their chance development. It is necessary that there should be competent medical botanists in the field taking notes, and the more the better. There is but one way to obtain this desideratum, and that is by establishing in every medical school a chair, where shall be thoroughly taught the elements of analytical botany, together with all that is known of the science in its medical relations. Let every graduate of our medical schools be competent to analyze plants, and indoctrinated with such medico-botanical facts as are now known, and we shall soon reap a rich harvest of reward. And this can very easily be done, without any additional time spent in medical education. This branch could be *more easily and more quickly taught* than any other, and would soon become exceedingly interesting to the large majority of students.

I can see no earthly reason why chemistry should stand so closely allied to medicine, and botany be entirely ignored. Try them by any reasonable test, and botany will prove itself the more valuable of the two. Which one has contributed the greater number of remedies to Materia Medica? From which shall we expect the greater supply in the future? How much of practical value is carried home by the student, after listening to a season's lectures by his professor of chemistry? The whole truth in regard to chemistry may be easily told. Its value to medicine is comparatively nothing in the *medical college.* It confers its benefits in the *laboratory*, out of sight and unseen of the student. Not one in ten thousand students ever acquire sufficient knowledge of chemistry to pursue it practically; and if he should do even this much, it is only as an abstract science, with rarely any practical medical value.

I do not wish to be understood as condemning the study of chemistry. I only mean that, as taught in our medical colleges, its value is exceedingly problematical. It is an abstract science; it deals in hidden and abstract truths and principles, and very few of our medical students ever comprehend it. It would be far otherwise with botany. From the very first lecture, the student could see and handle the objects of his study; and as he could see order and system arising out of the apparently confused mass of plants around him, his interest would be excited, and he could instantly apply everything told him, without any abstract or abstruse thought Nor would he ever be called on to deal in abstactions, until he had made such progress in the materialistic studies that the abstract ideas connected with the science would come as a natural sequence from the former, without effort or mental labor on his part. And I am very certain that a very large majority of medical students would pursue the study of botany with pleasure, and be practical analysts on graduation, fully prepared and able to gather up and enrich their profession with many now unknown botanical facts.

With this knowledge, every practitioner would soon find out all the medicinal plants which grew in his own neighborhood, and would more often avail himself of their use than he could possibly do without it. When he found an account of any plant in a journal, he could always know whether or not he could find it at home And should he discover any new treatment for any disease in the "*domestic practices*" of his neighborhood, he could not only determine the plant so used, but quickly find out all that is known about it by reference to authors, and be able in addition to communicate his own observations to the profession through some journal, with the certainty that he will be understood.

Some years since, I saw an article in a medical journal, very highly recommending a new remedy for puerperal convulsions. It was the ripe fruit or berries of *Solanam Carolinense;* but the author does not know the proper name, and described his remedy as the "common cow-nettle balls." His remedy would be known wherever that common name was known, but not elsewhere. With the proper botanical name, his essay could have conveyed valuable information to distant countries, and probably would have been extensively circulated. I will mention another but dissimilar case. Some years since I was told by a medical graduate that his Professor of Materia Medica had pointed out to him the common vine, so well known in our fields as the "May-pop," and told him that that was the "May-apple of the books." The similarity of the two common names had caused him to confound *Passaflora Incarnata* (May-pop, a worthless plant,) with the *Podophyllum Pettatum* (May-apple,) which is a valuable medical plant. But the worse feature of the case was that he taught his students to commit the same error. The natural conclusion is, that this professor did not know the real May-apple, although it was not uncommon in his neighborhood. And, bye-the-bye, I cannot well understand how any one can profess to teach Materia Medica, which deals largely in plants, without understanding *something* of botany.

What good reason, then, can be adduced why chemistry should hold its place and botany be excluded? Botany is certainly the more important, if for no other reason than because it is more practical, and will in the end give far more valuable results. I do not wish it understood that I advocate anything like appending botany to the chair of Materia Medica as a part of the duties of that professorship. I wish to see a separate and distinct professorship of *practical and medical botany* in every college; and I have not the least doubt but that it will repay the profession nearly as well as *any* other branch; and in saying this, I will not except even practice or surgery.

There are several questions still open, about which there is not now even a theory advanced, which it would be the special duty of this branch to investigate. What is now known as to the origin of the milk disease, prevalent in Kentucky some years since, and in all probability still numbering its victims, both human and bovine? Nothing, except that it is beyond a doubt of vegetable origin. But *what* plant, or whether any one in particular, is chargeable with the evil, no one now knows. The medical botanist who shall fully develop and elucidate the subject, will have well bestowed almost any amount of time and labor.

At one time the great Swedish botanist, Linnæus, while botanizing in Sweden, heard great complaint from the farmers that they lost great numbers of cattle every spring, when they turned them out into the woods. From his previous knowledge of the subject, he suspected that some poisonous plant eaten by the cattle caused the loss. He went into the woods in search of it, and soon pointed out a suspicious plant. The farmers set to work to eradicate it, and when this could not well be done, they enclosed it. The immediate result was that few other cattle were lost. Who shall do the same for the Kentucky scourge?

A theory has been somewhat discussed, attributing epidemic disease to a cryptogamous origin; and I confess a great partiality for it. Not because it has been proved true, but simply because no other explanation has ever been offered, adequate to explain the phenomena of epidemics. The vegetable origin of these diseases has not been proved; but there has been no one capable of making the investigation. There have been able botanists as there have been able physicians, but for the development of this subject, these two capacities must be united in one person; and there never has been such a union, and never will be, until botany is taught as a regular branch of medicine.

Several other questions of somewhat similar nature have suggested themselves to my mind, in thinking of the subject of medical botany, and I will mention some of them.

I have already stated that I thought there is good reason for believing that the presence of an umbelliferous inflorescence, is evidence that a plant possesses drastic powers. Will not irregularity in the structure of the plant indicate the same thing?—and the more the irregularity, the more decided is the drastic action? I use the term *irregularity* here in its common English acceptation, which includes all that is meant by the botanical terms—irregular, unsymmetrical, incomplete, imperfect and extra-axillary. The *Lobelia inflata*, the *Magnus Apollo* of our botanist friends, is an example of this principle. It is both irregular and unsymmetrical.

Is it not true that among the plants of every country a remedy is to be found for every disease that is native to that country? This thought is but a part of that corelation and coadaptation of parts which is known to obtain and hold good in every part of Nature.

Plants having bulbous, cormose and rhizomatose roots, are generally violent. Are they ever narcotic? Fibrous-rooted plants are not generally active, unless they have some irregularity in the flower. Coraline, subærial, tuberous and tap-rooted plants are usually innocent or mild in their effects.

In the study of these subjects, it must be borne in mind that plants deposit their active principles in three several parts—in the bark, in the root, and in the fruit; and that the active principle of every plant is the product of a true secretion, can be proved, I think, and will be the subject of another essay, if it is acceptable to this journal.

I hope that the reader will think over the subjects which I have suggested above in no narrow, sectarian spirit, but with a liberalism which will justify the expectation that no one will at least interpose any obstacle to this or any other attempt to advance medical knowledge, even if they should not join in the effort. It is a most difficult thing to demonstrate, beyond all question, the value of any untried measure, and I do not flatter myself that I have succeeded in this. But I may hope that I have awakened some interest in the medical study of botany, and I can but repeat my own decided conviction, that if such a chair as I have mentioned should be attached to our colleges, no man can predict the valuable advances which will thus be assured at no very remote future.

